# A statistical description of scattering at the quantum level

**DOI:** 10.1038/s41598-018-33425-8

**Published:** 2018-10-10

**Authors:** G. Laricchia, P. Van Reeth, S. E. Fayer, S. J. Brawley, R. Kadokura, A. Loreti, M. Shipman

**Affiliations:** 1 UCL Department of Physics and Astronomy, University College London, Gower Street, London, WC1E 6BT UK; 2 Present Address: Center for Fundamental Physics, Northwestern University, 2145 Sheridan Road, Evanston, IL 60208 USA

## Abstract

Quantum physics is undoubtedly the most successful theory of the *microscopic* world, yet the complexities which arise in applying it even to simple atomic and molecular systems render the description of basic collision probabilities a formidable task. For this reason, approximations are often employed, the validity of which may be restricted to given energy regimes and/or targets and/or projectiles. Now we have found that the lognormal function, widely used for the probability distribution of *macroscopic* stochastic events (as diverse as periods of incubation of and recovery from diseases, size of grains, abundance of species, fluctuations in economic quantities, etc.) may also be employed to describe the energy dependence of inelastic collisions at the quantum level (including ionization, electron capture and excitation by electrons, positrons, protons, antiprotons, etc.), by allowing for the relevant threshold energy. A physical interpretation is discussed in this article by analogy with the heat capacity of few-level systems in solid state physics. We find the generality of the analysis to extend also to nuclear reactions. As well as aiding the description of collision probabilities for quantum systems, this finding is expected to impact also on the fundamental understanding of the interface between the classical and quantum domains.

## Introduction

Ionization, excitation and electron capture are elementary processes in atomic and molecular collisions, also important in applications ranging from the modelling of the propagation of fast charged particles through matter (such as in plasmas and the atmosphere) to damage-control in medical dosimetry. The description of their energy dependence using quantum, classical and semi-classical approaches remains challenging, even in the case of simple atomic targets and structureless projectiles (e.g.^1–3^). Difficulties increase further in the case of higher-order processes (e.g. multiple ionization by multiply-charged projectiles) such that searches for empirical and semi-empirical scaling laws have been pursued over the past several decades (e.g.^4–8^).

Now we have found that the lognormal distribution, extensively employed in economics, industry, biology, ecology, geology, astrophysics (e.g.^9–11^), may also provide, to a good level of accuracy, the energy dependence of the probability for collision processes on the quantum scale for a variety of targets and projectiles, including electrons, positrons, protons and antiprotons. Examples are presented in this article and in the associated Online Supplementary Information (OSI). A physical interpretation of its significance in quantum systems is discussed and its applicability found to extend into the realms of nuclear reactions and solid state physics.

## Results and Discussion

The lognormal distribution is the continuous maximum-entropy probability distribution of a random positive variable whose logarithm is normally distributed. The probability density function is given by

1 $$f(x)=\frac{a}{x}\exp [-\frac{1}{2}{(\frac{{\rm{l}}{\rm{n}}(\frac{x}{{x}_{o}})}{b})}^{2}].$$

For the description of quantum scattering examined in this work, we define *x* to be equal to the reduced residual kinetic energy after a collision, namely $x=(E-{E}_{j})/{E}_{j}=E\text{'}/{E}_{j}$ where *E* is the projectile incident energy and *E*_*j*_ the threshold energy (*E*_*th*_) for a specific process $j$, for example ionization, electron capture, excitation, etc. (it should be noted that equally good fits are obtained if *x* is defined as equal to *E*′ but the above definition makes *x* dimensionless and consistent with the ln and exp functions in Eq. 1); *a*, *b* and *x*_*o*_ are fitting parameters with *b*^2^ being the variance of the corresponding normal distribution in (ln *x*) and *x*_*o*_ the median of *f*(*x*). The parameters give the maximum value of *f*(*x*) according to *f*(*x*_max_) = *a* exp*(b*^2^*/*2*)/x*_*o*_ where *x*_max_ = exp(ln*x*_*o*_ − *b*^2^).

In this article, we make use of the extensive availability of experimental data for electron-impact collisions and provide examples from ionization, excitation and molecular dissociation. We also include examples from positron-impact ionization and positronium formation as well as nuclear reactions induced by neutron- and proton- impact. Further examples (including ionization by protons and antiprotons as well as ionization and electron capture by multiply charged projectiles) are presented in the OSI. To avoid ambiguities, we present lognormal fits performed in all cases to experimental data only.

**Figure 1:**
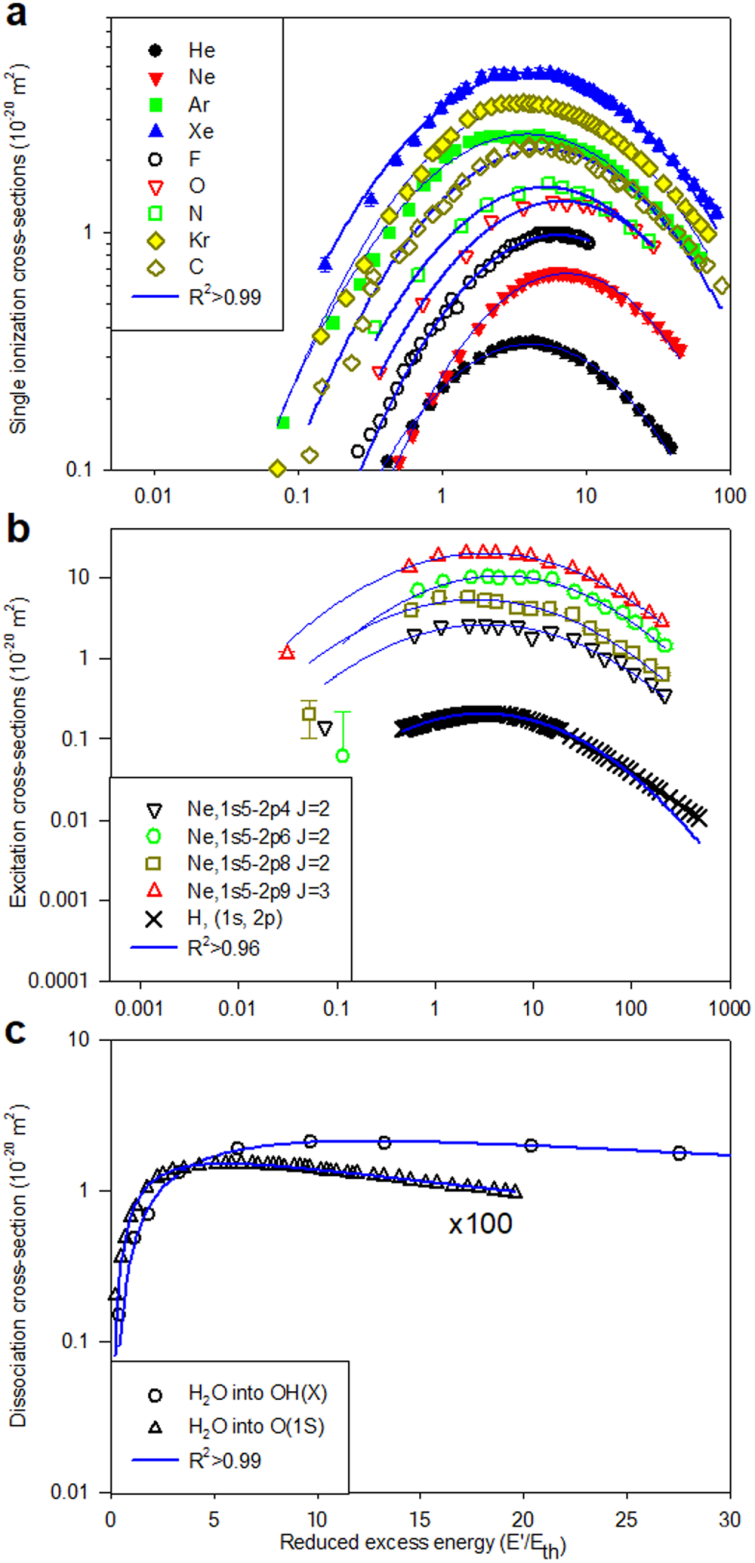
Ionization, excitation and dissociation by electron impact: comparison of measurements with lognormal fits (R^2^ values in the legends). (**a**) *Ionization*: inert (He, Ne, Ar, Kr, Xe)^12^ and 2^nd^ row atoms (C, N, O^13^; F^14^); (**b**) *Excitation*: H^16^ and Ne^17^; (**c**) *Dissociation* of H_2_O into O(^1^S)^18^ and OH(X)^19^ fragments. The cross section for O(^1^S) has been multiplied by a factor of 100 to aid comparison.

As a first case, we consider the single *ionization* cross-section by electron impact, the cross-section for a given process being a measure of the probability of that process resulting from a collision. Specifically in Fig. 1a, the results for the inert (He, Ne, Ar, Kr, Xe)^12^ and for second row atoms (C, N, O^13^; F^14^) are shown fitted to lognormals fits, yielding *R*^2^ > 0.990 in all cases except for argon which shows a slightly poorer fit (*R*^2^ > 0.988) due to a double peak structure apparent, to a lesser extent, also in krypton and xenon. (It is noted that structure around 10–20 eV also appears in the total cross-section for these atoms and is attributed to a d-wave *shape resonance*^15^). In Fig. 1b, examples from electron-impact *excitation* are presented. Specifically, the recommended values of the (1s-2p) cross-section for atomic hydrogen^16^ are displayed together with a lognormal fit (*R*^2^ > 0.996) as well as measurements^17^ for excitation out of the neon ${}^{3}P_{2}$ metastable level (*R*^2^ > 0.96). Examples of *molecular dissociation* into neutral metastable fragments are illustrated in Fig. 1c where the absolute cross-sections are shown for the production of O(^1^S)^18^ and OH(X)^19^ following electron impact on water. In both cases, lognormal fits to the data yield *R*^2^ > 0.99.

**Figure 2:**
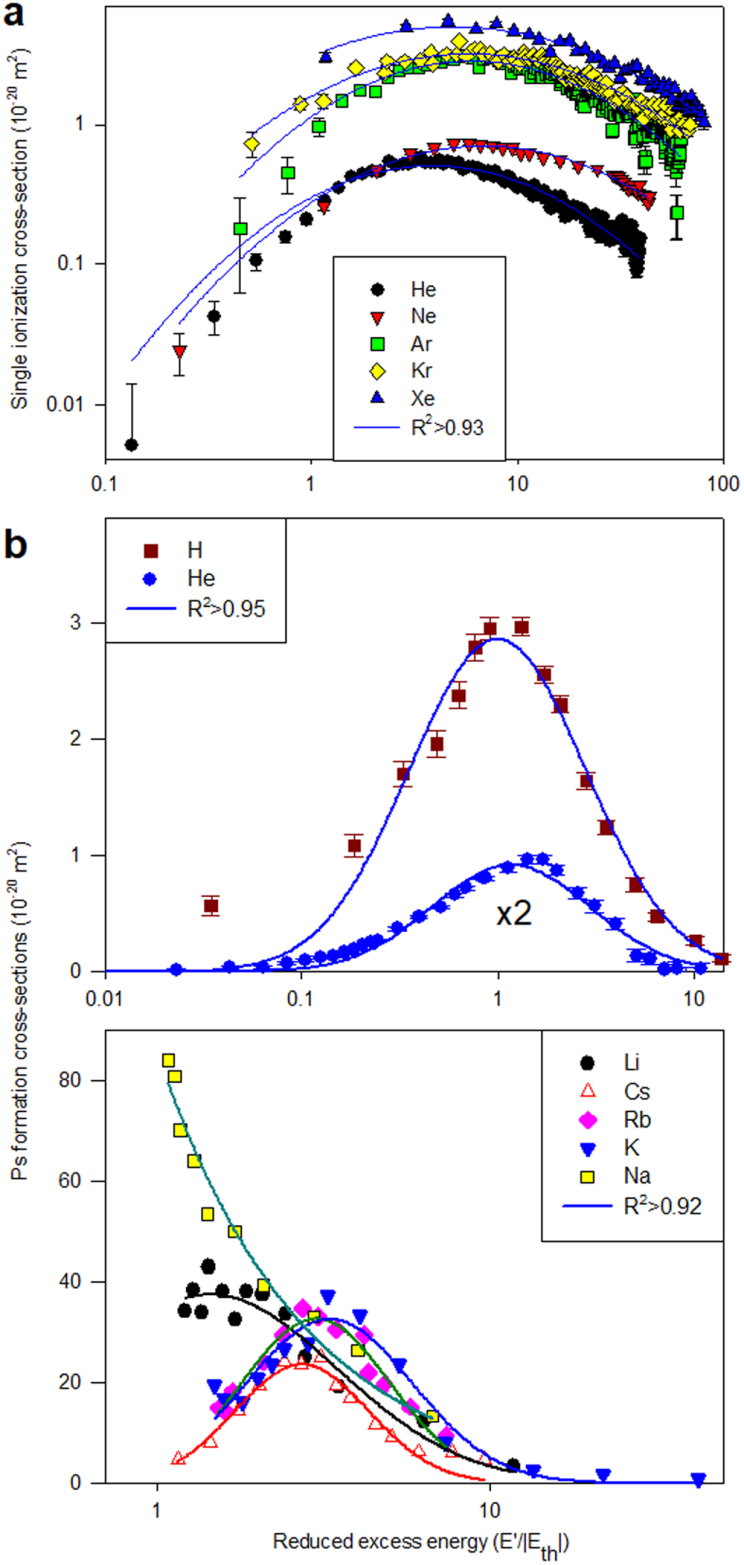
Ionization and electron capture by positron impact: comparison of measurements with lognormal fits (R^2^ values in the legends). (**a**) *Single ionization* for the inert atoms^20,21^; (**b**) *Positronium formation* cross sections for H^22^, He^20^ and the alkali metals^23–26^. The cross section for He has been multiplied by a factor of 2 to aid visual inspection.

In Fig. 2a, the analysis is extended to positron-impact single ionization cross-sections for the inert atoms where the data of^20,21^ are compared with lognormals (*R*^2^ > 0.93). We have found that the lognormal distribution also describes the energy dependence of rearrangement processes. In these cases, the projectile (e.g. a positron) can capture a target electron and form a bound state (e.g. positronium). Integral *positronium formation* cross-sections are shown in Fig. 2b. Results for e^+^  + H ^22^ and e^+^  + He ^20^ yield lognormal fits with *R*^2^ > 0.95. Determinations for the alkali metals^23–26^ are also consistent with lognormals (*R*^*2*^ > 0.92). It should be noted that Ps formation for these atoms is exothermic (i.e. *E*_*th*_ < 0) and the plots are thus shown versus (*E*′/|*E*_*th*_|).

Overall, we find the lognormal to describe with good accuracy the energy dependence of a variety of inelastic collisions over a broad energy range, e.g. for the electron impact excitation data in Fig. 1b from close to threshold to around *E*′*/E*_*j*_ ~100.

## Interpretation

As mentioned earlier, a multitude of *macroscopic* phenomena in nature may be described by lognormal distributions, including also the concentration of elements and their radioactivity in the Earth’s crust, size of raindrops and clouds, age of marriage, the galaxy mass density field, etc. (see e.g.^9–11,27,28^). What many of these phenomena may share is their genesis as the *product* of many independent random effects so that the use of the central-limit theorem applied to the logdata provides a justification for the use of statistical methods based on the normal distribution. That the energy dependence of quantum collision probabilities for a diversity of processes resulting from the impact of different projectiles on various targets may also be described by a lognormal distribution points to a plausible link, e.g. via correlations and collective effects which require treatments of electronic dynamics beyond the single active electron approach. Indeed these effects are known to be fundamental in atomic and molecular physics and pose a significant challenge to theoretical approaches^1–3^.

**Figure 3:**
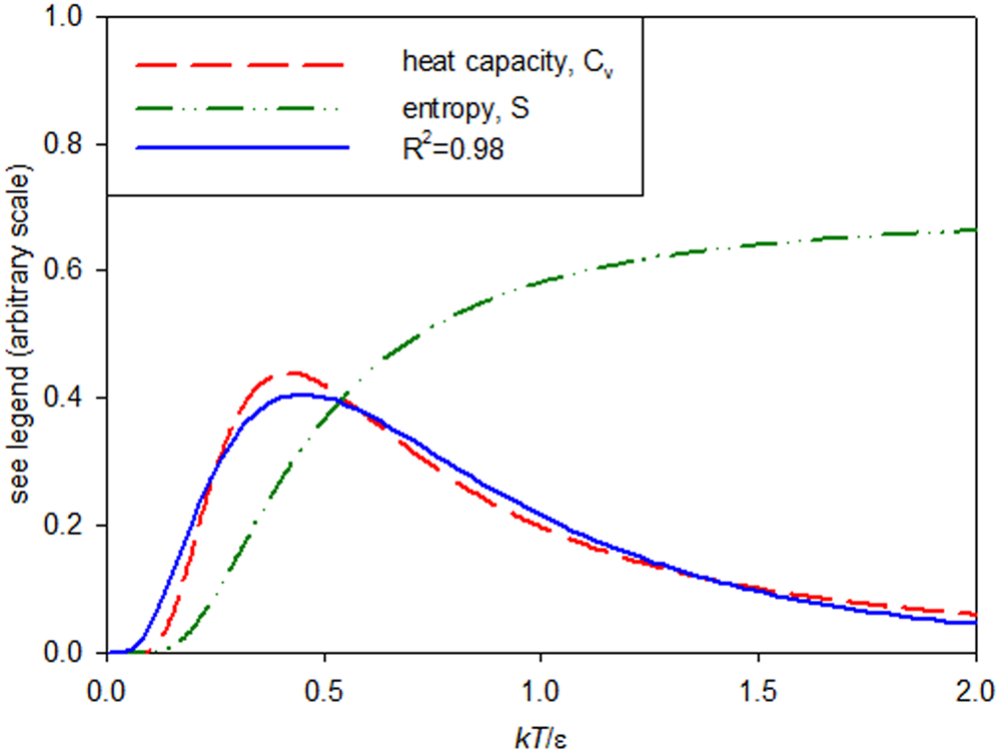
Heat capacity and entropy of a two-state system as a function of the energy *kT* and the energy gap (*ε*) between the two states (e.g.^29^). Also shown is a lognormal fit to *C*_*v*_.

The energy dependence of the cross-sections considered is reminiscent of that for the heat capacity of a few-level system in solid state physics (e.g. for paramagnetic salts)^29^. This dependence displays the so-called *Schottky anomaly* which, as illustrated in Fig. 3, may also be described by Eq. 1. (R^2^ > 0.982). The fundamental assumption in statistical thermodynamics favours that energy sharing which maximizes the number of accessible microconfigurations (${\rm{\Omega }}$, or distinguishable divisions of its energy content), so that physical systems tend to move towards maximal entropy (*S*) configurations over time, *S* being proportional to ln ${\rm{\Omega }}$. With reference to Fig. 3, initially with increasing energy, the entropy increases rapidly because the energy can be arranged in more ways over the microscopic degrees of freedom of the system. Above the maximum, where the accessible states are uniformly populated, the changes in entropy are small and consequently the heat capacity (${C}_{V}\equiv T(\partial {\rm{S}}/\partial T)$) decreases.

By analogy, during a collision when the atom is not in a stationary state, we may consider the atom (dominantly the electrons in the relevant atomic shell characterized by the threshold energy *E*_*j*_ for a specific process *j*) to be in thermal contact with the energy reservoir provided by the projectile kinetic energy, *E*. The atomic electrons are initially in the ground state (or some other eigenstate, as for excited neon in Fig. 1b). As the projectile approaches, it interacts with one (or more) electron(s) which can absorb energy through *virtual excitations* before relaxing into the final (eigen- or continuum-) state (e.g. excited, ionized, etc.). Each specific outcome into a final state *j* is then fully specified by the *total system energy*, *E*′, in the sense that at each *E*′ there is a definite value of the cross-section for the process *j* (whether endothermic or exothermic).

**Figure 4:**
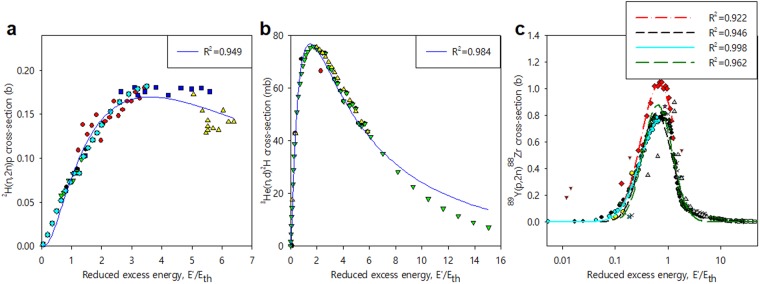
Nuclear reactions induced by neutron- and proton- impact: comparison of experimental cross-sections for (**a**) ^2^H(n,2n)p; (**b**) ^3^He(n,d)^2^H and (**c**) ^89^Y(p,2n)^88^Zr^30^ with lognormal fits (R^2^ parameters indicated in the legends).

Thus the lognormal may be considered as providing the energy dependence of the “background” cross-section which dominantly describes the *energy exchange* that results in the final state *j*. Of course, the cross-section may be modulated by quantum effects such as resonant or interference phenomena, examples of which were displayed in Fig. 1a and Figs S1 and S2. The generality of this interpretation, namely its independence from the details of the interactions at play, is reinforced by its applicability also to e.g. non-resonant nuclear reactions (see illustrations in Fig. 4^30^) and to the stopping of particles in matter (e.g.^31^).

As well as its intrinsic descriptive/predictive utility, the present finding is expected to impact also on the fundamental issue of the interface between the classical and quantum domains.

## Electronic supplementary material

Supplementary Information
