# Supplementary material for: A statistical description of scattering at the quantum level
Source: Sci Rep. 2018 Oct 10;8:15056. doi: 10.1038/s41598-018-33425-8 (PMC6180078; doi:10.1038/s41598-018-33425-8)
Supplement: Supplementary file 1 — Supplementary Information [file 41598_2018_33425_MOESM1_ESM.pdf]

A statistical description of scattering at the quantum level

G. Laricchia[1]*, P. Van Reeth[1], S. E. Fayer[1,2], S. J. Brawley[1], R. Kadokura[1], A. Loreti[1] and M. Shipman[1]

[1]UCL Department of Physics and Astronomy, University College London, Gower Street, London WC1E 6BT, UK.

[2] Present address: Center for Fundamental Physics, Northwestern University, 2145 Sheridan Road, Evanston, IL, 60208, USA.

*Corresponding author: g.laricchia@ucl.ac.uk

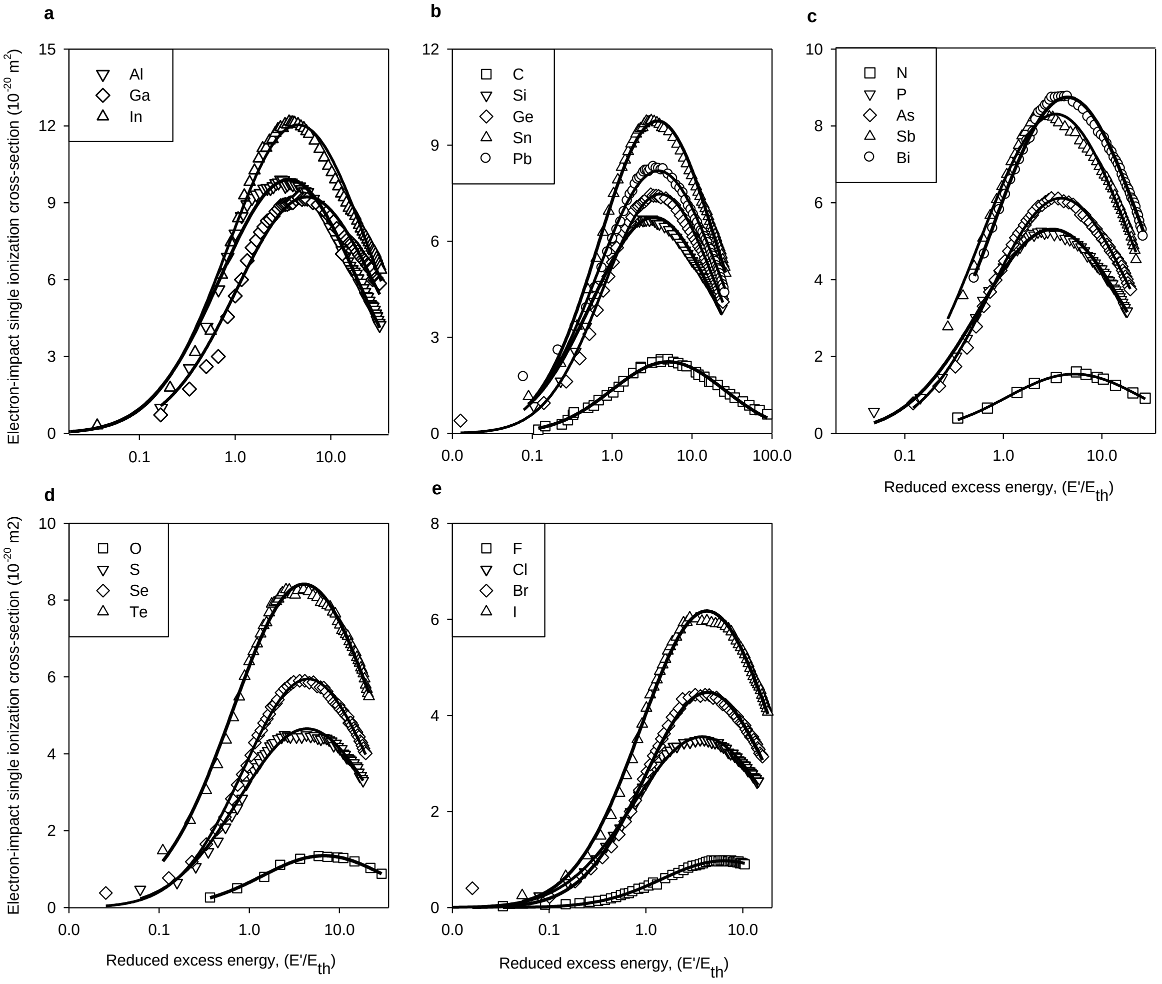

Fig. S1 Single ionization by electron impact on neutral atoms. **a**, Column III Al [1], Ga, In [2] (R^2>0.964). **b**, Column IV [1] except C [3] (R^2>0.976). **c**, Column V [1] except N [3] (R^2>0.986). **d**, Column VI [1] except O [3] (R^2>0.984). **e**, Halogens [5] (R^2>0.993).

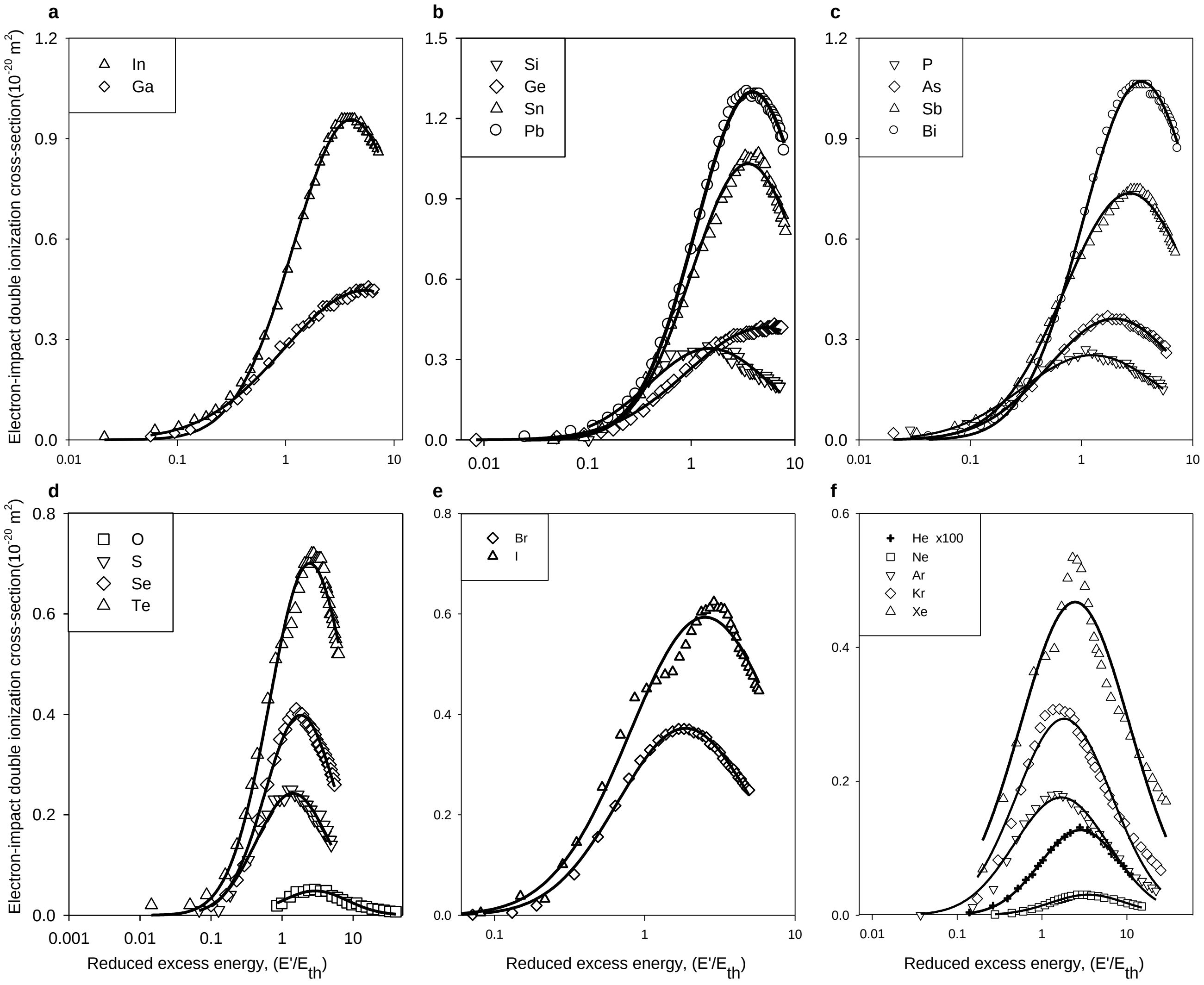

Fig. S2 Double ionization by electron impact on neutral atoms. **a**, Column III [2] (R^2>0.995). **b**, Column IV [1] (R^2>0.937). **c**, Column V [1] (R^2>0.995). **d**, Column VI [1] except O [4] (R^2>0.943).**e,** Halogen [5] (R^2>0.964). **f**, Noble gases [6] (R^2≥0.964). Iodine, tellurium and xenon have structure (to some extent visible also in Extended Data Figures 1d and 1e) which reduces the goodness of the fits. The structure has been associated with 4d type resonance [1], [7].

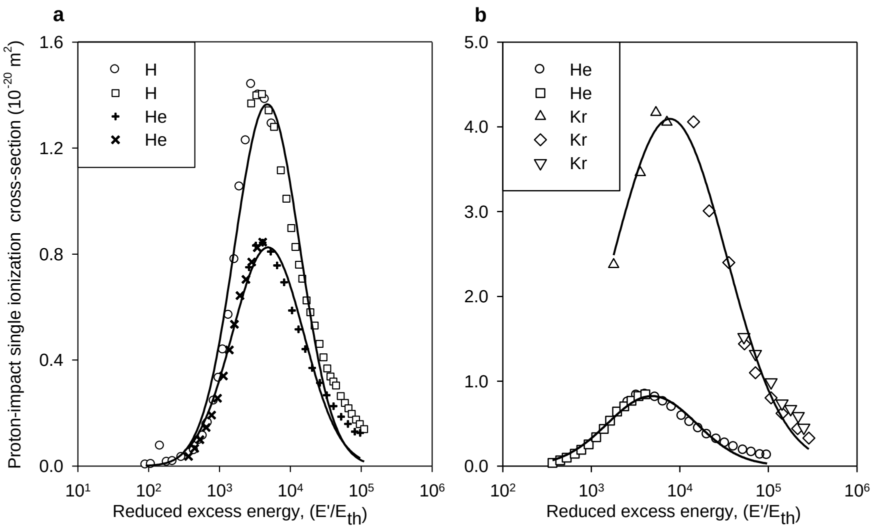

Fig. S3 Single ionization by proton impact. **a**, H: □ [8],○ [9] (R^2=0.953), He: + [10], × [11] (R^2=0.967). **b,** He: ○ [10], □ [11] (R^2=0.967),Kr: △ [12], ◇ [13], ▽ [14] (R^2=0.981).

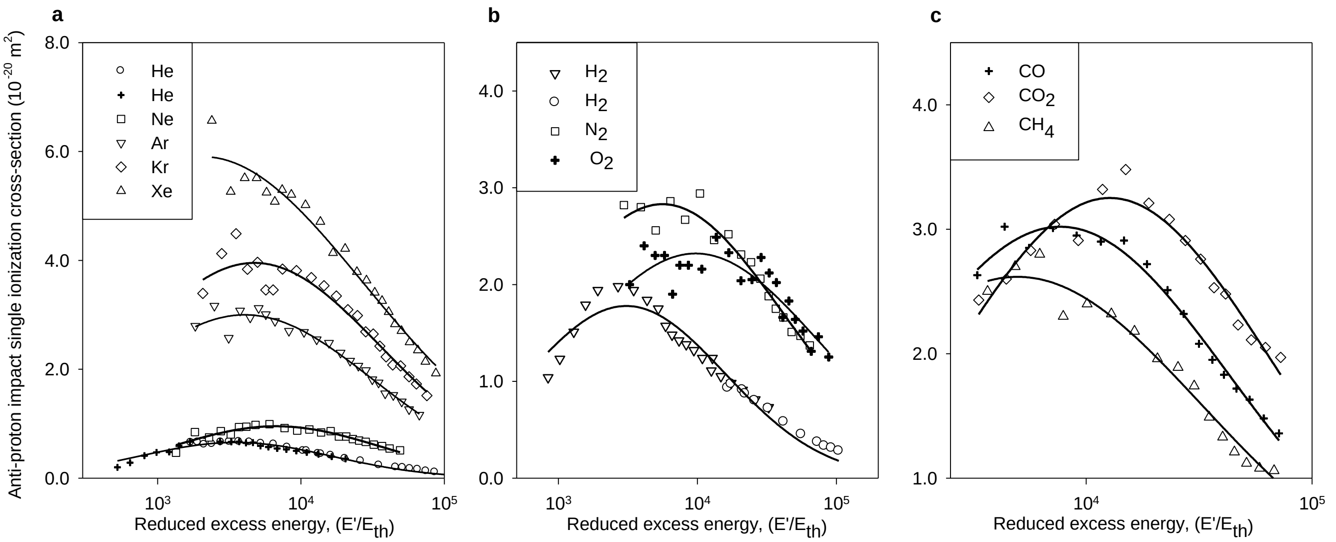

Fig. S4 Single ionization by anti-proton impact. **a**, He: + [15] , ○ [16], Ne: □, Ar: ▽, Kr: ◇, Xe: △ [17] (R^2>0.883). **b**, H_2: ▽ [15], ○ [18] , N_2: □ [19], O_2: + [20] (R^2>0.779). **c**, CO: +, CO_2: ◇, CH4: △ [19] (R^2>0.935).

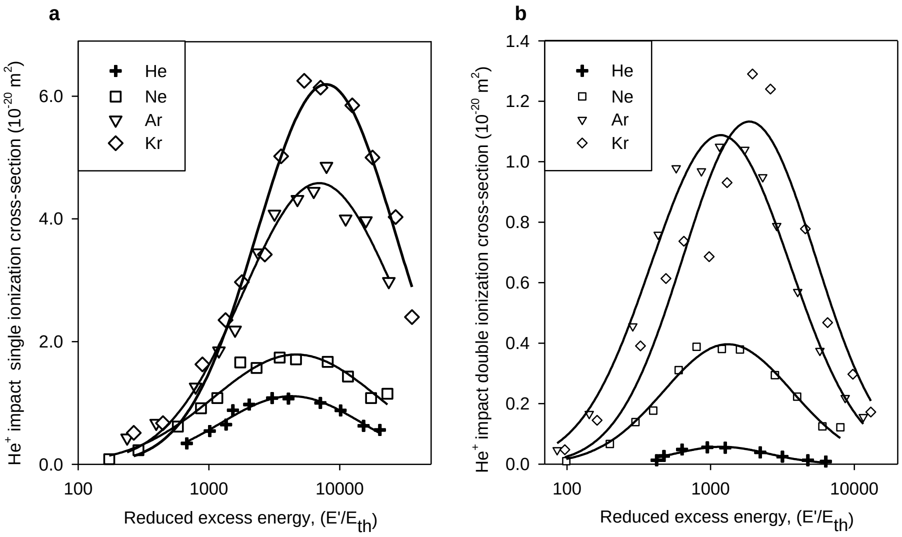

Fig. S5 Ionization by He^+ impact. **a,** Single ionization He: +, Ne: □, Ar: ▽, Kr: ◇ [21] (R^2>0.960). **b**, Double ionization by He^+ impact on Noble gases. He: +, Ne: □, Ar: ▽, Kr: ◇ [21] (R^2>0.908).

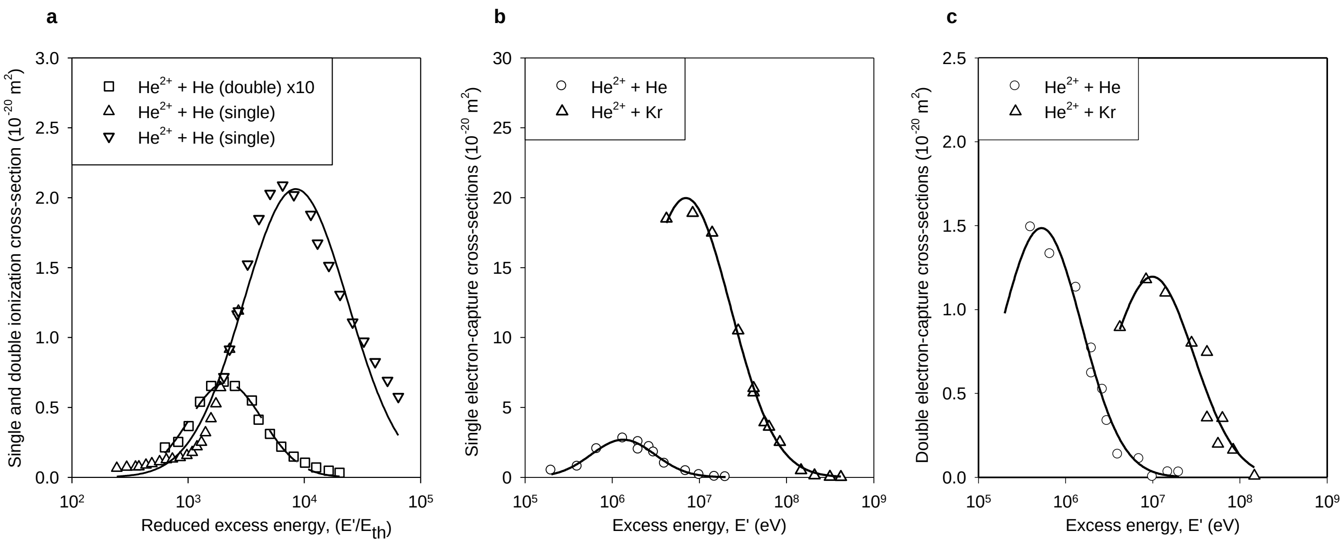

Fig. S6 Ionization and electron capture by He^{2+} impact. **a**, Single ionization of He: ▽ [10], △ [11] (R^2= 0.965); double ionization of He : □ [10] (R^2= 0.982). **b,** Single and **c**, double-electron capture from He and Kr [22] (symbols) (R^2>0.96 and >0.93, respectively). In b and c, the data are plotted versus E' since E_{th} =0 for double-electron capture from H.

.

References for Supplementary Information

[1] Freund R. S., Wetzel, R. C., Shul, R. J. & Hayes,T. R. Cross-section measurements for electron-impact ionization of atoms. *Phys. Rev. A* **41**, 3575-3595 (1990).

[2] Shul, R. J., Wetzel, R. C. & Freund, R. S. Electron-impact ionization cross-sections of the Ga and In atoms. *Phys. Rev. A* **39**, 5588 (1989).

[3] Brook, E., Harrison, M. F. A. & Smith, A. C. H. Measurements of the electron impact ionisation cross sections of He, C, 0 and N atoms. J. Phys. B: At. Mol. Phys. **11**, 3115 (1978).

[4] Thompson, W. R., Shah, M. B. & Gilbody, B. H. Single and double ionization of atomic oxygen by electron impact. J. Phys. B: At. Mol. Phys. **28**, 1321-1330 (1995).

[5] Hayes, T. R.,Wetzel, R. C. & Freund, R. S. Absolute electron-impact-ionization cross-section measurements of the halogen atoms. *Phys. Rev. A* **35**, 578-584 (1987).

[6] Rejoub, R., Lindsay, B. G. & Stebbings, R. F. Determination of the absolute partial and total cross sections for electron-impact ionization of the rare gases. *Phys. Rev. A* **65**, 042713 (2002).

[7] Younger, S. M. Giant resonance effects in the electron-impact ionization of heavy atoms and ions. *Phys. Rev. A* **35**, 2841 (1987).

[8] Shah, M. B. & Gilbody, H. B. Experimental study of the ionisation of atomic hydrogen by fast H^+ and He^{2+} ions. *J. Phys. B: At. Mol. Phys.* **14**, 2361-2377 (1981).

[9] Shah, M. B., Elliott, D. S. & Gilbody, H. B. Ionization of atomic hydrogen by 9–75 keV protons. *J. Phys. B: At. Mol. Phys.* **20**, 2481 (1987).

[10] Shah, M. B. & Gilbody, H. B. Single and double ionisation of helium by H^+, He^{2+} and Li^{3+}ions. *J. Phys. B: At. Mol. Phys.* **18**, 899-913 (1985).

[11] Shah, M. B., McCallion, P. & Gilbody, H. B. Electron capture and ionisation in collisions of slow H^+ and He^{2+} ions with helium. *J. Phys. B: At. Mol. Phys.* **22**, 3037-3045 (1989).

[12] DuBois, R. D., Toburen, L. H. & Rudd, M. E. Multiple ionization of rare gases by H^+ and He^+ impact. *Phys. Rev. A* **29**, 70-76 (1984).

[13] Dubois, R. D. Electron Production in Collisions between Light Ions and Rare Gases: The Importance of the Charge-Transfer and Direct-Ionization Channels. *Phys. Rev. Lett.* **52**, 2348-2351 (1984).

[14] Cavalcanti, E. G., Sigaud, G. M., Montenegro, E. C. & Schmidt-Böcking, H. Absolute cross-sections for multiple ionization of noble gases by swift proton impact. *J. Phys. B: At. Mol. Opt. Phys.* **36**, 3087-3096 (2003).

[15] Hvelplund, P. *et al.* Ionization of helium and molecular hydrogen by slow antiprotons. *J. Phys. B: At.Mol. Opt. Phys.* **27**, 925 (1994).

[16] Andersen, L. H. et al. Single ionization of helium by 40-3000 keV antiprotons. Phys. Rev. A **41**, 6536 (1990).

[17] Paludan, K. *et al.* Single, double and triple ionization of Ne, Ar, Kr and Xe by 30 - 1000 keV impact p− impact. *J. Phys. B: At. Mol. Opt. Phys.* **30**, 3951–3968 (1997).

[18] Andersen, L. H. et al. Non-dissociative and dissociative ionization of H_2 by 50–2000 keV antiprotons. *J. Phys. B: At. Mol. Opt. Phys.* **23,** L395 (1990).

[19] Knudsen, H. *et al.* Non-dissociative and dissociative ionization of N_2, CO, CO_2, and CH_4 by impact of 50–6000 keV protons and antiprotons. *J. Phys. B: At. Mol. Opt. Phys.* **28**, 3569-3592 (1995).

[20] Bluhme, H. *et al.* Non-dissociative and dissociative ionization of O_2 by impact of 40–1000 keV antiprotons. *J. Phys. B: At. Mol. Opt. Phys.* **30**, 3417 (1997).

[21] Dubois, R. D. Multiple ionization of He^+ - rare-gas collisions. *Phys. Rev. A* **39**, 4440 (1989).

[22] DuBois, R. D. Ionization and charge transfer in He^{2+} - rare-gas collisions. II. *Phys. Rev. A* **36**, 2585 (1987).
